# Magnetic resonance imaging and spectroscopy for differential assessment of liver abnormalities induced by *Opisthorchis felineus* in an animal model

**DOI:** 10.1371/journal.pntd.0005778

**Published:** 2017-07-14

**Authors:** Alexandra G. Pershina, Vladimir V. Ivanov, Lina V. Efimova, Oleg B. Shevelev, Sergey V. Vtorushin, Tatjana V. Perevozchikova, Alexey E. Sazonov, Ludmila M. Ogorodova

**Affiliations:** 1 Central Research Laboratory, Siberian State Medical University, Tomsk, Russia; 2 Department of Biotechnology and Organic Chemistry, National Research Tomsk Polytechnic University, Tomsk, Russia; 3 Center for Genetic Resources of Laboratory Animals, Institute of Cytology and Genetics SB RAS, Novosibirsk, Russia; Seoul National University College of Medicine, REPUBLIC OF KOREA

## Abstract

**Background:**

European liver fluke *Opisthorchis felineus*, causing opisthorchiasis disease, is widespread in Russia, Ukraine, Kazakhstan and sporadically detected in the EU countries. *O*. *felineus* infection leads to hepatobiliary pathological changes, cholangitis, fibrosis and, in severe cases, malignant transformation of bile ducts. Due to absence of specific symptoms, the infection is frequently neglected for a long period. The association of opisthorchiasis with almost incurable bile duct cancer and rising international migration of people that increases the risk of the parasitic etiology of liver fibrosis in non-endemic regions determine high demand for development of approaches to opisthorchiasis detection.

**Methodology/Principal findings:**

*In vivo* magnetic resonance imaging and spectroscopy (MRI and MRS) were applied for differential assessment of hepatic abnormalities induced by *O*. *felineus* in an experimental animal model. Correlations of the MR-findings with the histological data as well as the data of the biochemical analysis of liver tissue were found. MRI provides valuable information about the severity of liver impairments induced by opisthorchiasis. An MR image of *O*. *felineus* infected liver has a characteristic pattern that differs from that of closely related liver fluke infections. 1H and 31P MRS in combination with biochemical analysis data showed that *O*. *felineus* infection disturbed hepatic metabolism of the host, which was accompanied by cholesterol accumulation in the liver.

**Conclusions:**

A non-invasive approach based on the magnetic resonance technique is very advantageous and may be successfully used not only for diagnosing and evaluating liver damage induced by *O*. *felineus*, but also for investigating metabolic changes arising in the infected organ. Since damages induced by the liver fluke take place in different liver lobes, MRI has the potential to overcome liver biopsy sampling variability that limits predictive validity of biopsy analysis for staging liver fluke-induced fibrosis.

## Author summary

Opisthorchiasis caused by *Opisthorchis felineus* is a fish-borne parasitic worm infection spread in Russia and some European countries. The morbidities provoked by *O*. *felineus* infection are cholangitis and bile duct fibrosis. Long-term infection is associated with high risk of developing cholangiocarcinoma, a generally incurable bile duct cancer type. Due to lack of specific symptoms, *O*. *felineus* infection often escapes detection. Thus, opisthorchiasis diagnosis, especially in non-endemic regions, is a serious problem for physicians. In the paper, an animal model of *O*. *felineus* induced opisthorchiasis has been evaluated by *in vivo* magnetic resonance spectroscopy (MRS) and magnetic resonance imaging (MRI). Application of MR-techniques allowed to detect a characteristic MRI pattern of *O*. *felineus* infected liver, get valuable information about the severity of organ impairments, and bring to light some metabolic changes provoked by the helminth in the liver. Since damages take place in different liver lobes, MRI has the potential to overcome sampling variability of liver biopsy that limits liver fluke-induced fibrosis staging. The use of MR-techniques is very advantageous for investigating parasitic infection. Collection of experimental MR-data gives a new impulse to examination of infected humans and encourages to implement these methods in routine diagnosis of infections, including but not limited to opisthorchiasis.

## Introduction

Two liver fluke species of the genus *Opisthorchis*—*O*. *felineus* and *O*. *viverrini—*are known as human pathogenic agents. This fluke parasite inhabits the bile duct of the host liver, causing local mechanical damage and chronic inflammation, and having general toxic effects on the whole body [1]. Bile duct dilatation and mechanical obstruction accompanied by bile sludge formation are common complications of chronic opisthorchiasis [2]. Generally, liver fluke infection causes hepatobiliary pathological changes and leads to cholangitis, cholecystitis, and cholelithiasis.

The most severe complication of opisthorchiasis is malignant transformation. There is a significant association between cholangiocarcinoma (CAA) and *O*. *viverrini* infection; that liver fluke is considered carcinogenic (group I) to humans [3–5]. *O*. *felineus* is classified by IARC as group 3 due to limited experimental data [6]. However, in sources published in the Russian language, the association between CAA and chronic long-term *O*. *felineus* infection has been emphasized [7,8]. In whole, periductal fibrosis is considered an important risk factor for bile duct cancer and is associated with CCA development [9,10]. In the meantime, there is no significant association between liver fluke infection and cirrhosis, at least for closely related species *O*. *viverrini* and *Clonorchis sininsis* [5].

Chronic liver diseases, mainly liver fibrosis, represent a public health problem worldwide [11]. The generally discussed disease entities associated with liver fibrosis are nonalcoholic fatty liver disease (NAFLD), alcoholic liver disease, chronic viral hepatitis and chronic cholestasis, primary biliary cholangitis, and primary sclerosis cholangitis [12]. Fibrosis induced by liver flukes is commonly out of focus, though chronic infection caused by helminths of the genus *Opisthorchis* leads to periductal fibrosis and severe abnormalities in the hepatobiliary system [3].

It is clear that the pathogenesis of liver fibrosis depends on the underling etiology [13]. The main reason for complicated recognition of the cause of fibrotic or inflammatory process consists in the fact that liver responds to various injuries in a limited number of ways [14].

Parasitic liver flukes of the genus *Opisthorchis* are widespread in Eurasia—in Russia (mainly West Siberia), Ukraine, Kazakhstan (*O*. *felineus*), and South Asia (*O*. *viverrini*). In the endemic regions more than 750 million people are at high risk of liver fluke infection [5]. There are some reports about *O*. *felineus* infected people in EU countries [15,16]. It is very important to note that most infected persons, especially those with chronic infection (liver fluke lifespan may exceed 25 years [4]), show no symptoms at all or non-specific symptoms which are very difficult to recognize, so the infection is frequently neglected [17,18]. Taking into consideration increasing international migration of people, it is essential to bear in mind the risk of the parasitic etiology of liver fibrosis in the non-endemic regions [19]. In time, non-detected opisthorchiasis infection may be a cause of false diagnosis and complications in the treatment strategy of choice [17]. Furthermore, it is very important to exclude liver fluke infection, when assessing the transplantation potential of living liver donors.

Search for non-invasive tests for liver fibrosis diagnosis is the main trend in hepatology, since the “gold-standard” method—liver biopsy—has some shortcomings, such as invasiveness, complications, and sampling variability [11,13,20]. MR imaging-based techniques for liver fibrosis assessment are very promising and are being actively developed currently [21].

Magnetic resonance imaging (MRI) and magnetic resonance spectroscopy (MRS) share common physical background and are based on a nuclear magnetic resonance (NMR) phenomenon [22]. In the presence of a static magnetic field, for a nucleus of spin 1/2, absorption of energy emitted by a radiofrequency coil induces "flips" of a magnetic moment to another orientation. This "spin flip" places some of the spins in their high energy state. Relaxation processes which return nuclei back to the lower energy state after switching off the radiofrequency signal come with generation of a measurable amount of the radio frequency signal. The produced NMR signals received by the radiofrequency coil allow to generate MRS and MRI after Fourier transformation.

MRI has become an increasingly important imaging technique for investigating patients with hepatic and biliary disorders [23,24]. Although MRI examination is more expensive in comparison with computed tomography and ultrasound, this modality is widely used due to higher spatial resolution [25] and absence of ionizing radiation. MRS methods offer an opportunity to assess relative tissue metabolite concentrations based on the chemical shift phenomenon [26] and are very useful in clinical and biomedical studies for examining metabolic changes *in vivo* non-invasively [25,27]. In particular, 1H MRS is successfully used to determine relative lipid concentration in hepatic tissue [28], whereas 31P MRS allows to detect phosphorylated metabolites, including high-energy phosphates [25]. MRS methods are implemented to investigate the hepatic metabolic state and are applied as non-invasive diagnostic techniques for studying hepatobiliary pathology [29–31].

Despite the fact that MRI and MRS have become increasingly important imaging techniques for investigation of patients with liver and biliary disease [23,24], there are few papers describing application of *in vivo* MR-techniques for studying liver fluke infection [32–35] and no papers depicting the use of these methods for investigating *O*. *felineus*.

In this study we used magnetic resonance techniques (MRI/MRS) for differential assessment of liver abnormalities induced by *O*. *felineus* in an experimental animal model. We also found out correlations between the MR-findings and the histological data as well as the data of the biochemical analysis of liver tissue.

## Materials and methods

### Ethics statement

The experimental protocol was approved by the Bioethics Review Committee of the Institute of Cytology and Genetics, Siberian Branch, Russian Academy of Sciences, Novosibirsk (No. 30 from 20.11.2015). All animal experiments were conducted according to the principles of the Guide for the care and use of laboratory animals [36].

### Experimental opisthorchiasis model

Metacercariae of *O*. *felineus* were obtained from naturally infected fish (*Leuciscus leuciscus*) caught in the river in the endemic areas of Western Siberia (the Tom, Tomsk), Russia. A permit was not required to collect these fish according Federal law of the Russian Federation No. 166 “About fisheries and conservation of water biological resources” from 20.12.2004. For the experiment, 5-week-old male hamsters (n = 8) (*Mesocricetus auratus*) were infected intragastrically with 50 metacercariae per hamster, according to the previously described protocol [37]. Age-matched intact male hamsters (n = 8) were used as controls. The hamsters were housed two in a cage (OptiRAT) under conventional conditions and were permitted ad libitum access to food and water. The animals were handled in pathogen-free environment. At 8 weeks post-infection, the hamsters were scanned with MRI and MRS *in vivo*. Following the MR examinations, the subsets of the infected and intact animals were deeply anesthetized with carbon dioxide and euthanized by decapitation. Blood and liver samples were collected for examination from each hamster from the control (n = 8) and infected (n = 8) groups. Serum and blood analyses were performed using routine procedures (Methods in S1 Text).

### MRI and MRS *in vivo*

All 1H/31P MR experiments were performed on a horizontal tomographic scanner with magnetic field intensity of 11.7 T (Bruker, Biospec 117/16 USR, Germany). Prior to MR examinations, the animals were fasted overnight. The animals were anaesthetized with gas anesthesia (Isofluran; Baxter Healthcare Corp., Deerfield, IL) using a Univentor 400 Anesthesia Unit (Univentor, Zejtun, Malta). The animal body temperature was maintained with a water circuit installed into the table bed of the tomographic scanner, which maintained the temperature of 30°C on its surface. A pneumatic respiration sensor (SA Instruments, Stony Brook, NY) was placed under the lower body part, which allowed to control the anesthesia depth.

#### MRI

All MR images were recorded with a receiver—transmitter 1H volume coil (T11440V3). High-resolution T2-weighted images of the hamster liver (section thickness 1 mm; field of vision (FOV) 4.0 × 4.0 mm; matrix 512 × 512 dots) were acquired with respiratory triggering using TurboRARE (Rapid Imaging with Refocused Echoes) method with pulse sequence parameters: TE_eff_ = 18 ms, TR = 900 ms, Flip Angle = 180°, RARE Factor = 4. Serial images in axial and coronal orientation were recorded for each animal.

#### 1H/31P-MRS

Proton and phosphorus spectra were recorded with a receiver—transmitter double-tuned 1H/31P surface coil (T11619V3). The coil was positioned to the right upper abdominal quadrant of the hamster liver in the right lateral decubitus position, which allowed to minimize the signal capture by muscles and decrease movement artifacts. Before each session, recording of 3 orthogonal packages of liver sections (Multi-slice Tri-Pilot scanning) was conducted, to guide the correct coil positioning relative to liver tissue (in case of infected hamster to capture liver damaged zone). The protons spectra were recorded using the spatially localized PRESS (Point Resolved Spectroscopy) method with TE = 20 ms, TR = 2.5 ms, and 256 replicates, voxel size 5×5×5 mm. Water signal in the spectra was suppressed by the variable power and optimized relaxation delays (VAPOR) method.

The phosphorus spectra were recorded using the non-spatially localized single-pulse method with TE = 3 ms, TR = 100 ms, and 2,048 replicates. The spectrum range was 40 ppm, which made it possible to obtain information about the tissue contents of 5 substances represented by 7 peaks, phosphomonoester (PME; 6 ppm), inorganic phosphate (Pi; 5 ppm), phosphodiester (PDE; 2 ppm), creatine phosphate (PCr; 0 ppm originated from muscle contamination), and nucleoside triphosphate (NTP; 2.7, 7.8, and 16.5 ppm).

#### MRS data processing

Time domain data were Fourier transformed after Gaussian multiplication (center, 0 ms; width, 30 ms) and phase corrected. Quantification of the spectral peak areas was performed using the TOPSPIN 2.0 PV software package (Brucker), including polynomial baseline correction followed by frequency domain curve peak integration. The 1H and 31P metabolite concentrations were calculated from the peak areas and expressed relative to an area under the curve. Intracellular pH was calculated based on the chemical shift difference δ between Pi and α-NTP (δ = f_Pi_-f_αNTP_-7.56) using the Henderson-Hasselbalch equation [38]:

pHi=6.75+log10[(δPi−3.27)/(5.69−δPi)] (1)

### Histological analysis

Liver samples (from posterior segments of the right lobe) were fixed in 10% buffered formalin, and embedded in paraffin. Tissue sections were cut into 4–5 μm-thick slices and stained with hematoxylin and eosin. Histological analysis was performed with the optical microscope Axiostar plus (Carl Zeiss, Germany). For differential staining of collagen in the liver samples, Van Gieson’s method was used. Fibrosis was graded according to METAVIR score [39]. Hepatic steatosis was graded on a 0–3 scale through visual estimation of the percentage of hepatocytes containing intracellular vacuoles of fat [40].

### Liver tissue analysis

Two samples of liver tissue were taken from posterior segments of the right lobe and medial segments of the left lobe for each animal. The samples were divided into five pieces in accordance with subsequent examinations and immediately placed in liquid nitrogen. Homogenization was performed using Tissue Grinders (PELLET PESTLE^®^ Cordless Motor, Kimble Chase, TN) on ice.

#### Lipids assay

Extraction of lipids from 200 mg of liver tissue (two pieces for each animal) was performed using Folch method [41]. The concentrations of cholesterol, triglycerides and phospholipids in the lipid extract were determined spectrophotometrically (SF2000, Russia) using Chronolab kits (Spain). The total lipids were gravimetrically determined by drying 2 ml of liver lipid extract in a glass vial.

#### ATP assay

60 mg of liver tissue (two pieces for each animal) was homogenized in 1 ml of 3% trichloroacetic acid and centrifuged at 4,000 g, 4°C, 20 min. The pH of the supernatant was adjusted to 7.4 with 1 M Tris. The ATP concentration was measured on Anthos lucy2 (Asys Hitech GmbH, Austria) using the Adenosine 5′-triphosphate (ATP) Bioluminescent Assay Kit (Sigma-Aldrich, St. Louis, MO).

#### Protein assay

30 mg of liver tissue (two pieces for each animal) was homogenized in 400 μl of NP-40 buffer (150 mM sodium chloride, 1.0% Triton X-100, 50 mM Tris, pH 8.0) and centrifuged at 12,000 g, 4°C, 20 min. Then the protein concentration was determined in the supernatant using Bradford assay [42]. Bovine serum albumin (BSA fraction V, Sigma) was used as a protein standard.

#### Glycogen assay

20 mg of liver tissue (two pieces for each animal) was homogenized in 1 ml of 0.3 M HClO_4_. The homogenate was incubated with aminoglucosidase (AG, Sigma) in 50 mM sodium acetate, 0.02% BSA, pH 5.5 for 2 h at RT, and centrifuged at 10,000 g for 1 min. The amount of the released glucose was determined by the Hexokinase Method [43] using the Glucose Reagent (Vector-Best, Russia). Glucose (Sigma) was used as a standard. Free glucose in the homogenate was measured without addition of AG and subtracted from AG-treated samples.

#### Western blot

20 mg of liver tissue was homogenized on ice using dounce homogenizer in 1 ml of lysis buffer (50 mM Tris-HCl (pH 7.5), 250 mM sucrose, 5 mM sodium pyrophosphate (NaPPi), 50 mM NaF, 1 mM EDTA, 1 mM dithiothreitol (DTT), 0.5 mM phenylmethylsulfonyl fluoride (PMSF)) supplemented with Mammalian Protease inhibitor Cocktail (P8340 Sigma). The protein concentration was quantified by Bradford assay, using BSA (Sigma) as a standard. Following lysis sodium dodecyl sulfate (SDS) was added to a final concentration of 0.2% and samples are boiled for 5 min. The samples (twenty micrograms of total proteins) were loaded onto a 15% SDS-PAGE gel according to Laemmli protocol [44]. The separated proteins were transferred to nitrocellulose membranes. The membranes were blocked with 3% non-fat milk in Tris-buffered saline (TBS) with 0.1% Tween 20 and 50 mM NaF for 1 h at room temperature and probed overnight at 4°C with primary antibodies in 5% BSA supplemented with 50 mM NaF, followed by incubation with Goat Anti-Rabbit IgG H&L (HRP) (ab6721) (Abcam, USA) in TBS supplemented with 5% milk and 50 mM NaF for 1 h at room temperature. The membranes were developed with a ECL Chemiluminescent Substrate Reagent Kit (Thermo Scientific, USA) and scanned by G:box XT4 (Syngene). The band intensities were quantified using GeneTools (Synegene). The primary antibodies Phospho-AMPKα (Thr172) (CellSignaling technology, Inc.) and Anti-beta Actin antibody (ab8227) (Abcam, USA) were used.

### Statistical analysis

Statistical analyses were performed using IBM SPSS Statistics for Windows, Version 21.0 (Armonk, NY: IBM Corp.). Data were presented as median (range) for data with non-normal distribution and mean (SD) for data with normal distribution. The data were tested by the Shapiro—Wilk test for normality. The differences among continuous variables with normal distribution were analyzed by the t-test and among continuous variables with non-normal distribution—by the Mann-Whitney test. For correlation analyses, the Person (for continuous variables) and Spearman (for ordinal variables) correlations were used. The P value below 0.05 was considered as significant.

## Results

### Histological analysis

**Fig 1:**
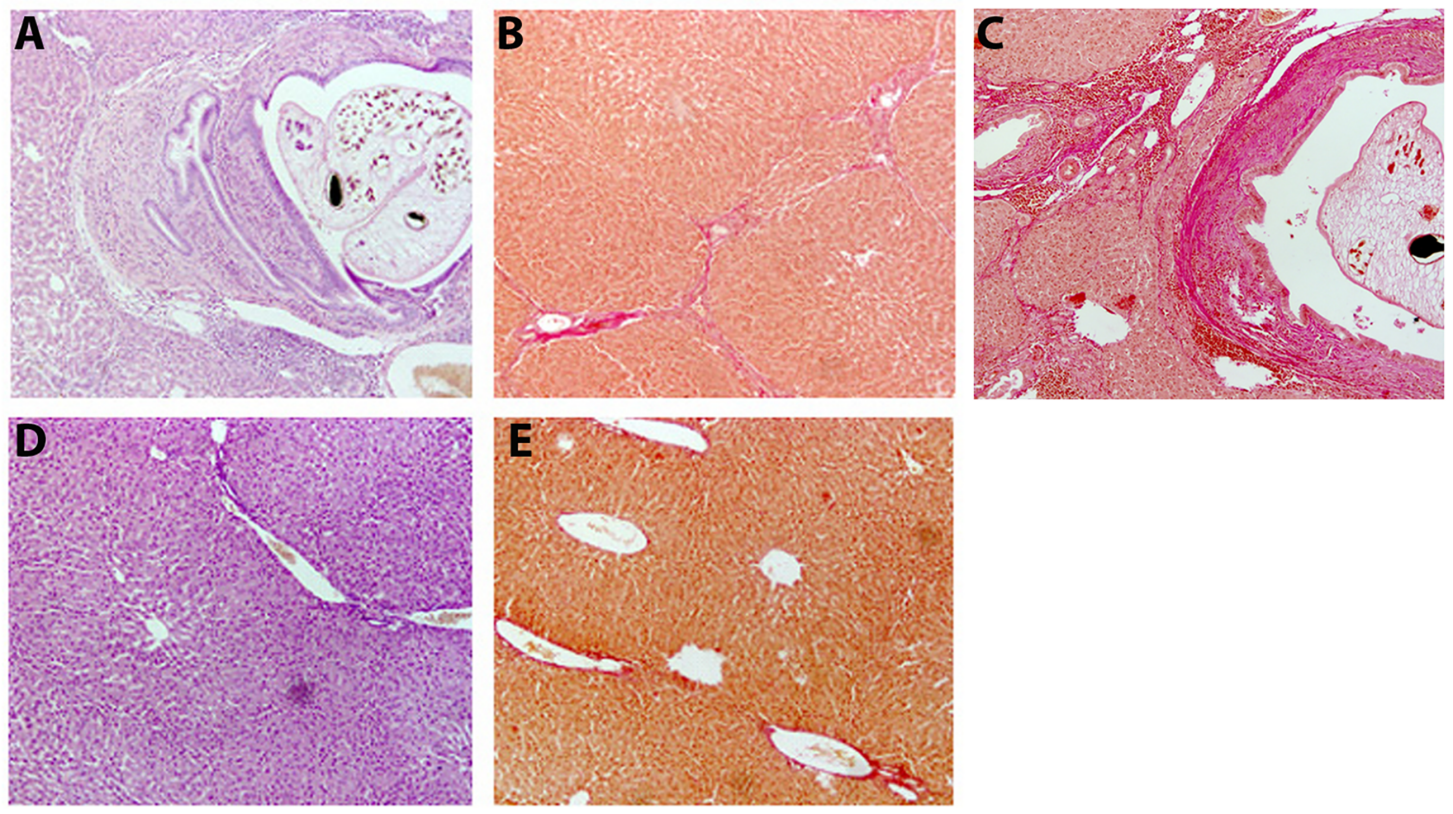
Histological analysis of hamster liver. (A) Microphotographs of *Opisthorchis felineus* infected hamster liver at 8 weeks post-infection. An adult fluke is found in bile duct lumen. Small bile duct proliferation and chronic cholangitis accompanied by inflammatory infiltration in the bile duct wall; hematoxylin-eosin staining, ×100. (B) Mild portal fibrosis with thin septa formation (portal to portal and portal to central) and (C) pronounced periductal fibrosis with chronic inflammation around the bile duct are observed. In adjacent hepatic tissue, fibrosis with porto-portal septa formation takes place. Van Gieson’s staining showing extensive deposition of mature collagen fibers, ×100. (D) Microphotographs of the uninfected hamster liver. No pathological changes are observed. Hematoxylin-eosin staining, ×100 and (E) Van Gieson’s staining, ×100.

Histological analysis data showed that *O*. *felineus* infection resulted in derangement of the liver architecture, whereas the trabecular pattern mostly remained intact. Hepatocytes had various sizes and patchy exhibited cloudy swelling. Cholangitis in the portal tract was accompanied by pronounced periductal and mild portal fibrosis as well as focal cystic dilatations of the intrahepatic bile ducts. Small bile ductular proliferation and irregular periportal (mostly moderate) infiltration of inflammatory cells, with predominant population of lymphocytes and histiocytes, occurred (Fig 1). Fibrosis was irregular; patchy, incomplete, thin fibrous septa (portal to portal and portal to central bridge) were observed (Fig 1A, 1B and 1C). In whole, chronic cholangitis (mild to moderate inflammatory activity, A1-A2 according to METAVIR score) and mild chronic hepatitis were diagnosed, and the stage of fibrosis varied from 1 to 2 according to METAVIR score. There were no lipid droplets in the tested liver samples. No pathological changes took place in the liver in the reference group (Fig 1D and 1E).

### Blood and serum analysis

The results of blood and serum analyses are given in Results in S1 Text and Table in S1 Table. In the infected group of hamsters the levels of alanine aminotransferase (ALT) and gamma-glutamyl transferase (GGT) increased markedly. Significant elevation of cholesterol, triglycerides, and low-density lipoproteins (LDL) in serum of the infected animals was detected. All the above listed serum parameters correlated with the fibrosis stage. The concentration of albumin in serum of the infected hamsters was lower than in the reference group and was accompanied by a statistically significant increase in the urea concentration. However, there was no correlation between these parameters (r = -0.271, p = 0.309).

### Biochemical analysis of liver tissue

The liver to body index increased in the infected animals, while the spleen to body index remained unchanged (S1 Table). The results of liver tissue biochemical analysis are given in Table 1. There was a statistically significant rise in the cholesterol level in opisthorchiasis, and the cholesterol to phospholipid ratio also elevated in the infected liver. Total lipid content, triglycerides and phospholipids as well as glycogen did not differ between the control and infected groups of hamsters. A significant decrease in the protein concentration occurred in the infected livers. However, the calculated total protein content in the entire liver did not differ between both control and experimental groups due to enlargement of the organ in the infected hamsters. It is important to note, that infection did not lead to lowering of the ATP concentration in liver tissue.

**Table 1:** Liver tissue biochemical analysis.

Metabolite^#^	Control		Infected		Correlation with fibrosis stage	
	mean	SD	mean	SD	r	p-level
Protein, mg g^-1^	120.80	17.19	94.92*	15.62	-0.720	0.002
Total cholesterol, mg g^-1^	1.80	0.25	2.64*	0.59	0.781	0.000
Triglycerides, mg g^-1^	2.84	0.39	3.29	1.23	0.282	0.290
Phospholipids, mg g^-1^	9.89	0.84	8.93	1.05	-0.447	0.082
Total lipids, mg g^-1^	42.77	4.91	39.88	4.38	-0.136	0.615
Triglycerides to phospholipid ratio	0.26	0.04	0.32	0.09	0.463	0.071
Cholesterol to phospholipid ratio	0.37	0.06	0.60*	0.10	0.885	0.000
ATP, nmol g^-1^	2.77	0.66	2.94	1.04	-0.032	0.905
Glycogen, mg g^-1^	4.17	3.41	6.11	2.16	0.152	0.573

^**#**^Concentration calculated per gram wet weight of tissue

*difference between the infected and the control group is significant at p<0.01

r, correlation coefficient; ATP, Adenosine triphosphate.

### ^1^H MRI

**Fig 2:**
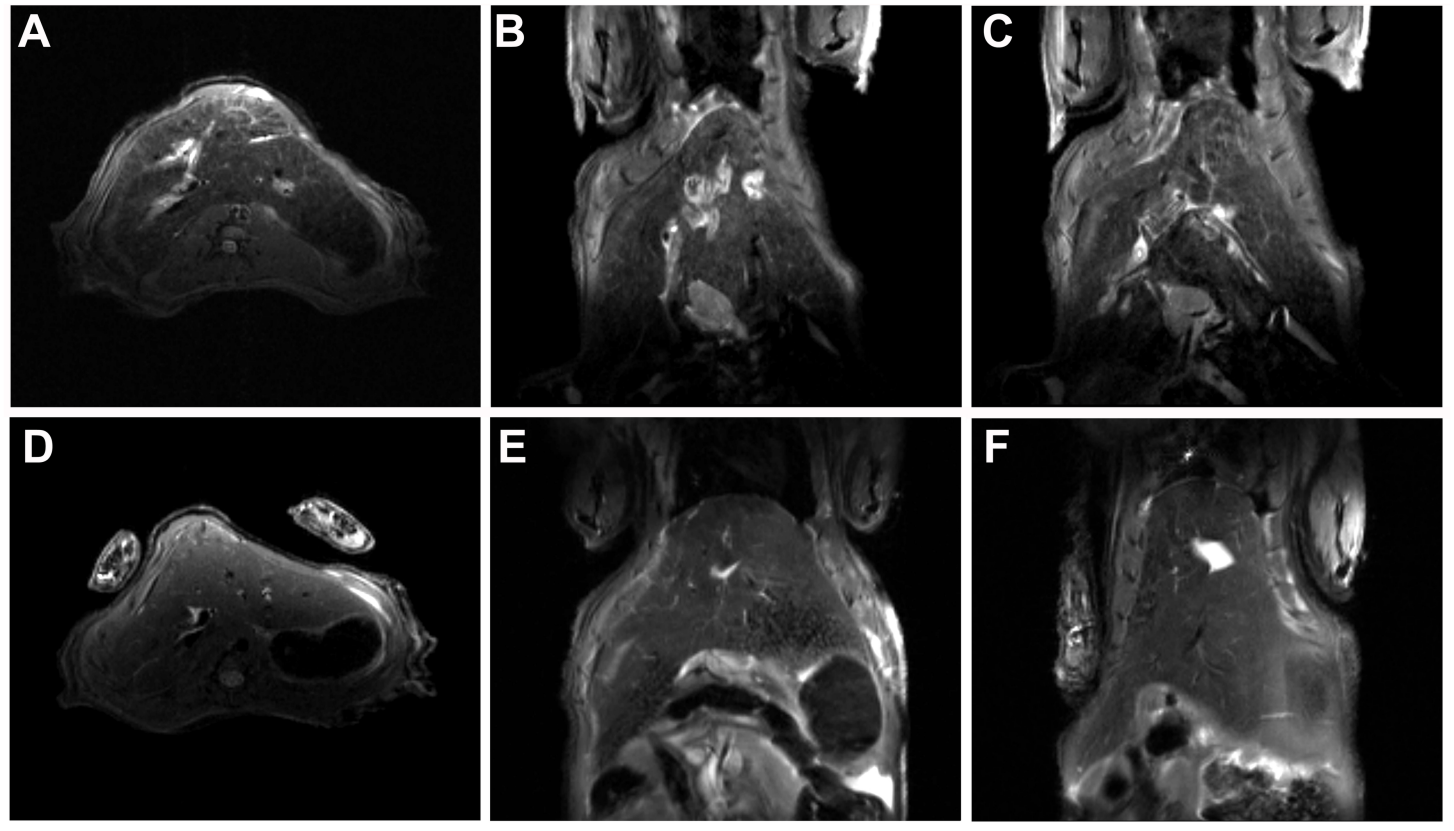
MRI of hamster liver. T2-weighted (A) axial and (B, C) coronal images of *O*. *felineus* infected liver and (D) axial and (E, F) coronal images of uninfected hamster liver (control) at 11.7 T. TurboRARE (Rapid Imaging with Refocused Echoes) with respiratory triggering, with pulse sequence parameters TE_eff_ = 18 ms and TR = 900 ms, Flip Angle = 180°, RARE Factor = 4. Bruker, Biospec 117/16 USR, Germany. In *O*. *felineus* infected hamster liver, the hyperintense signals from the bile duct wall as well as the bright zones extending from the dilated bile duct due to inflammation and fibrosis are clearly depicted. There are no pathological changes in the liver of the control hamsters.

A T2-weighted image of hamster liver infected with *O*. *felineus* for 8 weeks displayed pronounced bile duct abnormalities (Fig 2A, 2B and 2C). Hyperintense areas extended around the bile duct are found in different lobes of the liver in all infected for 8 weeks hamsters. The most common MR findings included periductal enhancement, intrahepatic bile duct dilatation, and bile duct wall thickening. We detected a hyperintense zone extending from the dilated bile duct due to inflammation and fibrosis, without pronounced peripheral bile duct enhancement. In the control group the liver parenchyma was homogenous and showed no dilatation of the bile ducts (Fig 2D, 2E and 2F).

### 1H MRS

**Fig 3:**
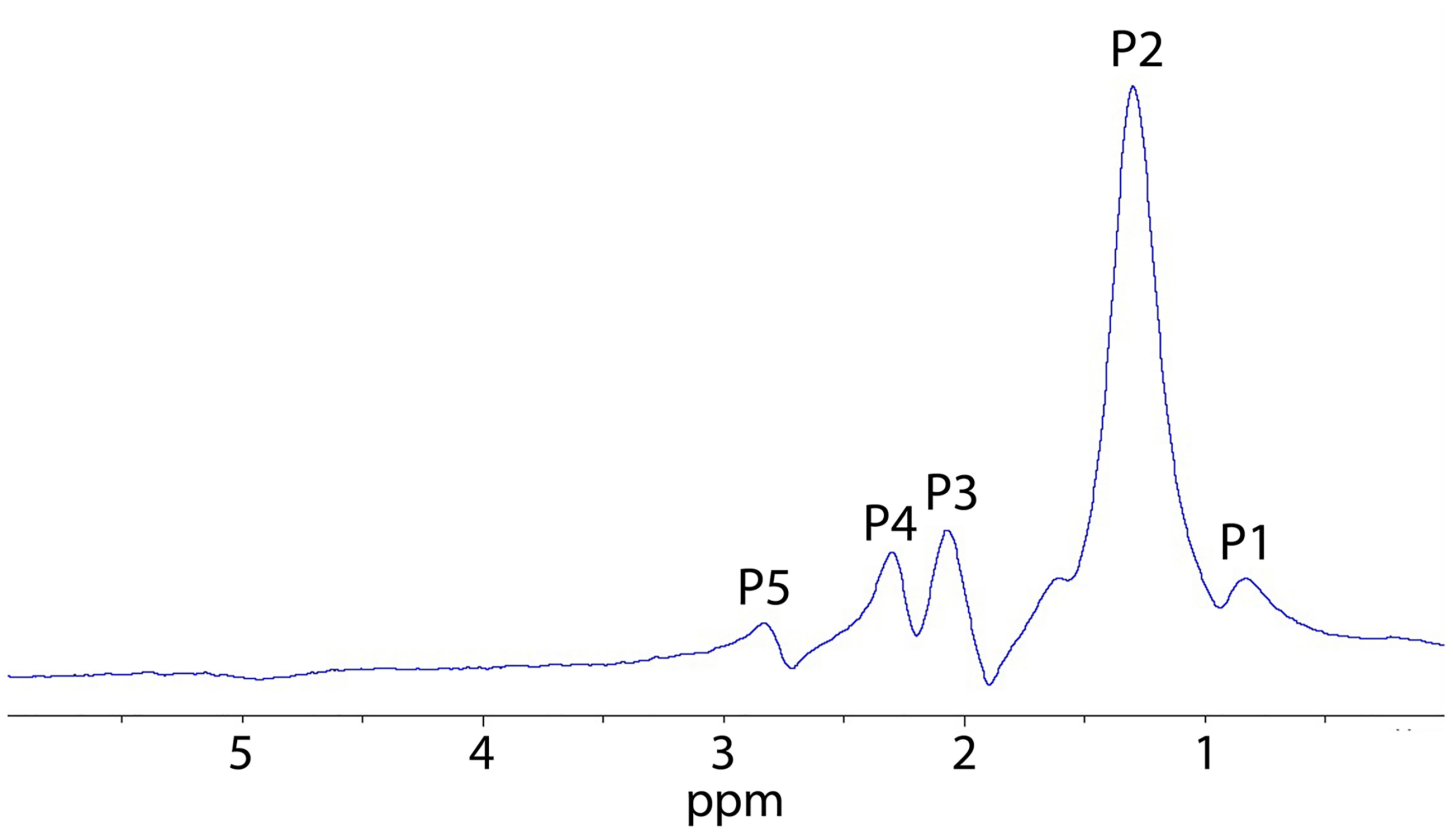
Typical *in vivo* 1H magnetic resonance spectrum of hamster liver. Spectrum was recorded using the spatially localized PRESS (Point Resolved Spectroscopy) method with TE = 20 ms, TR = 2.5 ms, 256 replicates, voxel size 5×5×5 mm. Water signal in the spectra was suppressed by the variable power and optimized relaxation delays (VAPOR) method. Bruker, Biospec 117/16 USR, Germany. Lipid resonance peaks are labeled: P1: methyl proton C**H**_3_ (0.9 ppm); P2: methylene (–C**H**_2_–)_n_ (1.3 ppm); P3: -C**H**_2_-CH = CH- (2.0 ppm); P4: -C**H**_2_-COO- (2.2 ppm); P5: -CH = CH-C**H**_2_-CH = CH- (2.8 ppm).

Analysis of the proton spectra (Fig 3) showed no statistically significant differences in the content of lipid compounds in the livers of the control and infected hamster groups (Table 2).

**Table 2:** Levels of hepatic lipid metabolites (*in vivo* 1H MRS).

	0.9 ppm	1.3 ppm	2.0 ppm	2.2 ppm	2.8 ppm	3.2 ppm	5.3 ppm
Infected	0.09	0.73	0.08	0.05	0.02	0.02	0.01
SD	0.03	0.09	0.03	0.04	0.02	0.02	0.01
Control	0.10	0.73	0.08	0.04	0.02	0.01	0.01
SD	0.03	0.06	0.02	0.02	0.01	0.02	0.02
Correlation coefficient with fibrosis stage	-0.250	0.026	0.062	0.042	0.041	-0.038	0.041
p-level	0.351	0.924	0.821	0.877	0.881	0.889	0.880

We found no correlation between the concentration of lipids in liver tissue determined according to biochemical analysis and certain characteristic peak areas in *in vivo* 1H MRS spectra. While the triglyceride and cholesterol concentrations, measured by biochemical analysis of liver tissue, correlated with 2.2 to 0.9 ppm peak ratio (r = 0.561, p = 0.041 for both), the triglyceride level correlated with 2.8 to 0.9 ppm peak ratio (r = 0.537, p = 0.032).

In fact, a peak at 0.9 ppm is attributed to protons in the methyl group of cholesterol, phospholipids and triglycerides, whereas peaks at 2.2 ppm and 2.8 ppm are assigned to protons bonded with carbons alpha and polyunsaturated carbons of triglycerides [45] [46]. More specifically 2.8 ppm/0.9 ppm peak area ratio is associated with a polyunsaturated bond [47,48].

Thus, according to the 1H MRS study, there were no significant changes in the lipid profile in the liver of the infected hamsters.

### 31P MRS

**Fig 4:**
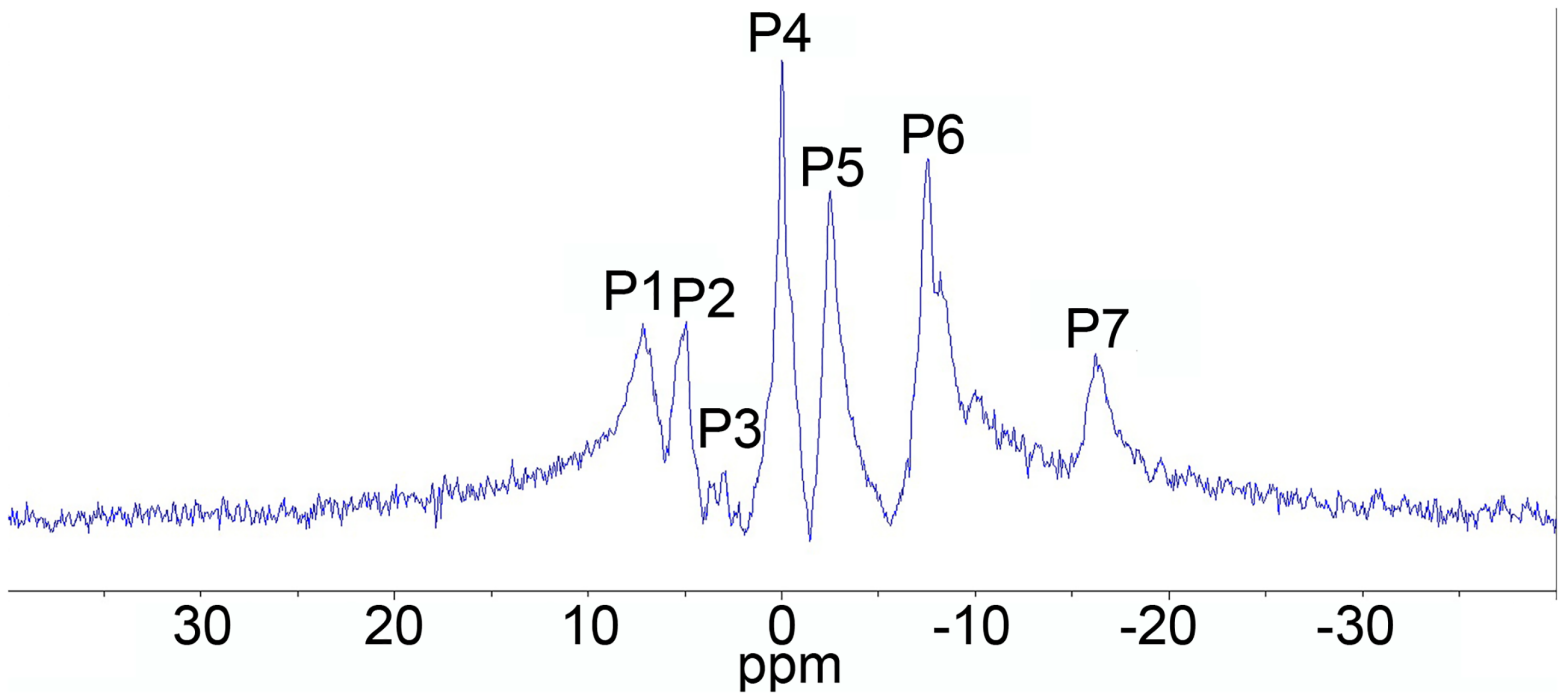
Typical *in vivo* hepatic 31P magnetic resonance spectrum of the right upper abdominal quadrant (includes liver, abdominal wall, subcutis, etc.) of a hamster. Spectrum was recorded using the nonspatially localized, single-pulse method with TE = 3 ms, TR = 100 ms, 1048 replicates. Bruker, Biospec 117/16 USR, Germany. Phosphorylated metabolites resonance peaks are labeled: P1: phosphomonoester (PME); P2: inorganic phosphate (Pi); P3: phosphodiester (PDE); P4: creatine phosphate (PCr); P5: NTP-γ; P6: NTP-α; P7: NTP-β.

Fig 4 presents typical 31P MR spectra of the hamster liver. The calculated fractions of various phosphorylated metabolites are given in Table 3. Analysis of phosphorous spectra showed that the hepatic PME level increased in the infected group of hamsters, while the PDE levels remained unchanged. Significantly lower levels of NTP-α were detected in the infected group of hamsters, whereas the peak area attributed to other high-energy compounds, such as NTP-β and NTP-γ, in the livers of the control and infected hamsters had no statistically significant differences. PME and NTP-α peak areas correlated with the fibrosis stage (r = 0.710, p = 0.002 and 0.726, p = 0.001, respectively).

**Table 3:** Levels of hepatic phosphorylated metabolites (*in vivo* 31P MRS).

	PME	P_i_	PDE	NTPγ	NTPα	NTPβ	pH_i_	NTPγ/ NTPα	NTPγ/ NTPβ	NTPβ/ NTPα	PME/PDE	Pi/NTPα	Pi/NTPβ	Pi/NTPγ
Infected	0.17**	0.09	0.04	0.11	0.24**	0.17	7.22	0.47	0.71	0.70*	4.45	0.38**	0.58	0.82*
SD	0.03	0.02	0.01	0.02	0.02	0.03	0.11	0.08	0.31	0.13	1.38	0.09	0.28	0.14
Control	0.13	0.08	0.04	0.12	0.28	0.15	7.23	0.42	0.94	0.53	3.80	0.28	0.55	0.67
SD	0.02	0.01	0.01	0.01	0.02	0.02	0.11	0.03	0.30	0.11	1.22	0.027	0.12	0.10
r	0.710	0.292	0.165	-0.279	-0.726	0.480	-0.031	0.337	-0.570	0.622	0.301	0.745	-0.107	0.645
p-level	0.002	0.273	0.541	0.296	0.001	0.060	0.909	0.202	0.021	0.010	0.257	0.001	0.693	0.007

r, Correlation coefficient with the fibrosis stage; PME, Phosphomonoesters; Pi, Inorganic phosphate; PDE, Phosphodiesters; NTP, nucleoside triphosphate, pH_i_, Intracellular pH.

*difference between the infected and the control group is significant at p<0.05.

**difference between the infected and the control group is significant at p<0.01.

Correlation analysis revealed that PME and NTP-α peak areas significantly correlated with serum ALT, AST, cholesterol, HDL, cholesterol to phospholipids ratio in liver tissue as well as with the fibrosis stage (S2 Table).

NTP-γ to NTP-α as well as NTP-γ to NTP-β ratios did not differ, whereas NTP-β to NTP-α ratio markedly increased in the infected group. Moreover, NTP-β/NTP-α ratio correlated with the fibrosis stage (r = 0.622, p = 0.010), cholesterol content (r = 0.622, p = 0.005) and cholesterol to phospholipids ratio in the liver (r = 0.664, p = 0.005).

The calculated intracellular pH did not differ in the control and infected groups.

### Western blot analysis

**Fig 5:**
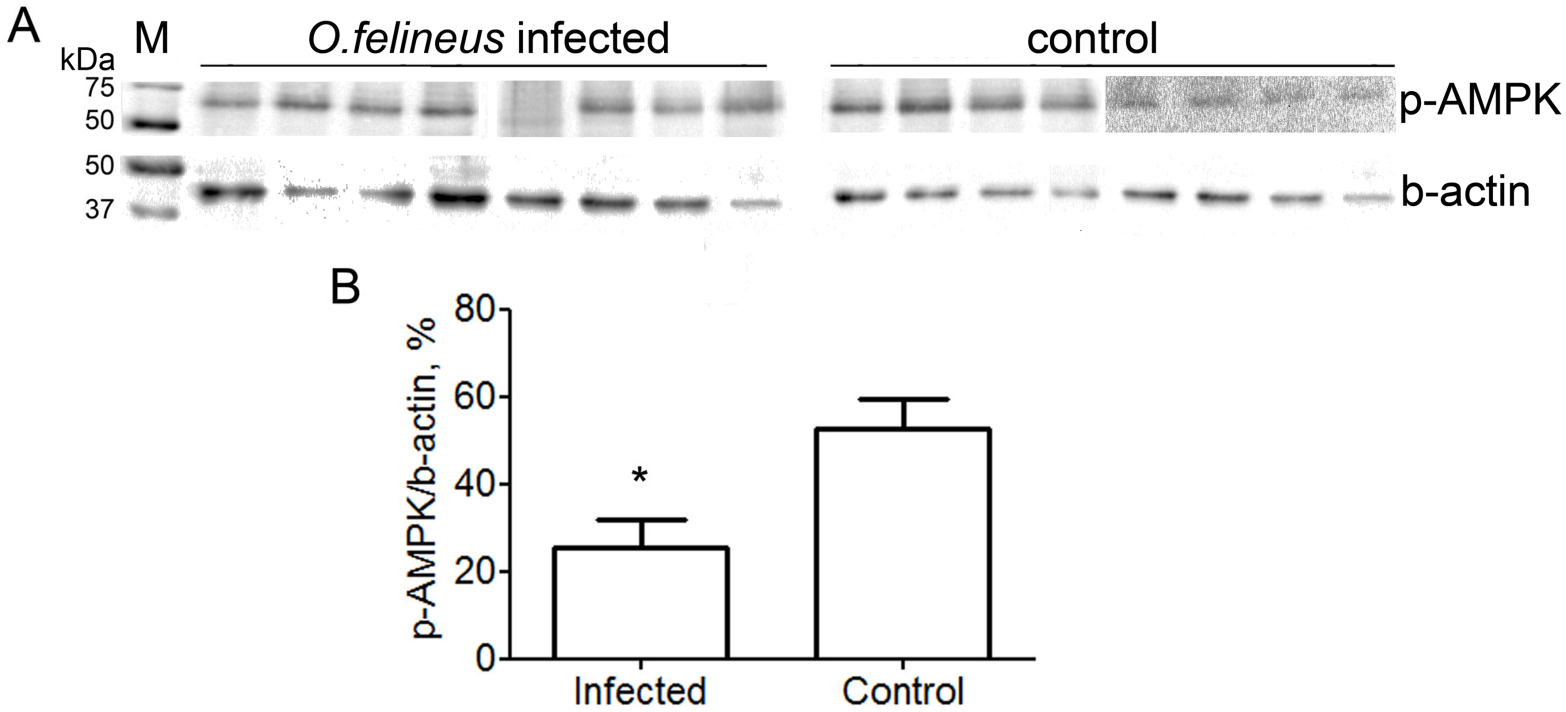
Analysis of the AMPK phosphorylation level in the hamster liver. (A) Western blots of (p-AMPK) phospho-AMPK and (b-actin) beta-actin in *O*. *felineus* infected and healthy (control) hamster liver; (M) Precision Plus Protein Western C Standards (BioRad) with Precision Protein StrepTactin-HRP Conjugate (BioRad) are used as a molecular weight marker. (B) The histogram depicts the level of phospho-AMPK determined through densitometry relative to beta-actin band. The values are represented as means±SD (n = 8 animals per group), *p<0.05 denotes significant differences between the infected and the control groups.

5’-AMP-activated protein kinase (AMPK) has been implicated in the control of hepatic glucose and lipid homeostasis, including fatty acid and sterol synthesis. It has been proposed to act as a ‘metabolic master switch’ mediating cellular adaptation to environmental or nutritional stress factors [49]. As illustrated in Fig 5 the amount of AMPK, phosphorylated by Thr172 residue of α-subunit (phospho-AMPK), decreased in *O*. *felineus* infected liver. This means that the activity of kinase in the infected liver is lower than that in the healthy one. Statistical analysis showed that the relative levels of phospho-APMK in the hamster liver correlated with the NTP-α level, determined according to 31P MRS, (r = 0.585, p = 0.028), cholesterol (r = -0.726, p = 0.003) and triglycerides (r = -0.590, p = 0.026) concentrations in serum as well as the fibrosis stage (r = -0.607, p = 0.021).

## Discussion

*O*. *felineus* infection leads to significant pathological changes in the bile duct and liver of the hamsters, which results in chronic cholangitis and pronounced periductal/mild portal fibrosis. The key biochemical marker of hepatic parenchymal cell injury is the observed 3-fold increase in ALT in serum of the infected hamsters occurring due to the release of the enzymes from liver tissue into the circulation. The ALT level elevation in opisthorchiasis has been emphasized by several authors [50,51]. The observed hepatomegaly in the infected hamsters, a common sign of parasitic infection [52], is caused rather by the inflammatory process in the liver [53,54]. The lowering of the protein concentration in liver tissue (per gram) of the infected animals may indicate an impaired protein synthesis function of hepatocytes. In the meantime, there is no difference in the total protein content calculated per whole liver between the infected and intact hamsters. Consequently, physiological adaptation to a metabolic demand as a driving mechanism of liver enlargement in the infected hamsters cannot be excluded [55].

*O*. *felineus*-induced liver lesions are clearly identified by MRI. The key MRI finding observed in our study is the pronounced intrahepatic biliary ductal dilatation with extension of T2-hyperintense damaged zone around the dilated bile duct. These spreading hyperintense zones correspond to fibrosis in adjacent to the bile duct hepatic tissue clearly identified by the histological analysis. The observed hyperintense signal from the bile duct wall is related to the inflammatory process diagnosed in the bile duct according to the histopathalogical study.

It is important to emphasize the difference of the *O*. *felineus* MRI-pattern from that of closely related liver fluke infections. Choi et al. [33] described that the hallmark of radiologic findings in clonorchiasis is diffuse, mild dilatation of the intrahepatic biliary tree, especially the peripheral bile ducts, without any evidence of obstruction. In our study the dilatation was sufficiently pronounced. Yet, MRI in humans may slightly differ from MRI in animal models due to differences in size of the organ. At the same time, the T2-weighted image of *O*. *felineus* infected liver demonstrated wider and more pronounced hyperintense zones in liver tissue around the dilated bile duct, compared to the *O*. *viverrini* infected liver [34]. The origin of these differences is probably related to the fact that *O*. *felineus* causes more pronounced liver damage, as opposed to *O*. *viverrini* [56]. Therefore, the development of an approach to differential diagnosis of *O*. *felineus* infection from other helminth infections requires further investigation in human population.

There is an additional reason for implementing of MRI for opisthorchiasis diagnosis. Since bile duct dilatation and T2-enchancement have taken place in different liver lobes of the infected individuals, liver biopsy sampling variability rises and limits predictive validity of biopsy analysis for staging liver fluke-induced fibrosis. In comparison with microscopic stool examination, MRI is a more expensive technique for diagnosing *O*. *felineus* infection, but it allows clinicians to evaluate opisthorchiasis-related liver damage [57]. Moreover, MRI can be very helpful for opisthorchiasis detection in infected people in non-endemic countries.

Morphological and functional damage of the liver that resulted from mechanical, inflammatory, and toxic injury may lead to disturbance of the metabolic processes in the liver.

Our 1H MRS data indicated no changes in the lipid profile of the liver and no fat droplets were observed in the liver of the infected hamsters according to histological examination. At the same time, biochemical analysis of hepatic tissue showed the elevated cholesterol concentration in liver tissue. Since MR techniques are sensitive only to molecules with a high degree of rotational molecular motion, the observed lipid resonances arise only from the intracellular fat droplets, whereas membrane lipids are not detectable using MRS [48]. Thus, the combination of MRS and biochemical data allows us to assume that cholesterol in the liver accumulates in the cellular membrane and the cholesterol to phospholipids ratio rises, respectively. This process may lead to loss of fluidity of liquid-crystalline domains in the cell membrane [58]. In turn, an increase in membrane rigidity may affect conformational freedom of integral membrane proteins and, consequently, their function. Therefore, the function of canalicular membrane transporters of cholesterol can be impaired even more, leading to enhancement of cholesterol excretion breakdown [59].

Observed in our study hyperlipidemia in combination with the increased cholesterol concentration and elevated cholesterol to phospholipid ratio in liver tissue indicate the disturbance of lipid metabolism in *O*. *felineus* infection. Alteration of lipid metabolism in parasite infection is emphasized by several authors [60–62]. For example, rise in the cholesterol/phospholipid ratio in liver plasma membrane and hyperlipidemia accompanied by strong accumulation of lipids in hepatic tissues during ancylostomiasis has been reported [60,63]. In case of *O*. *felineus* infection, cholesterol accumulates in the liver, however, no triglyceride and phospholipid accumulation occurs. It is little known about the main source and proportion of nutrients for *O*. *felineus* [37,64], but liver flukes inhabiting the bile ducts are likely able to uptake nutrients from the bile. Young et al. reported [65] that closely related species *O*. *viverrini* and *C*. *sinensis* transcribe genes encoding enzymes and accessory proteins directly involved in processing of bile constituents, and can utilize free lipids. Notably, these genes are absent in blood flukes and tapeworms.

Generally, cholestasis and biliary injury are associated with marked abnormalities of cholesterol metabolism and pronounced elevations in serum cholesterol and LDL [66,67]. Here mechanisms that can lead to cholesterol accumulation in liver tissue should be pointed out. At first, injury of epithelial bile duct cells can result in cholesterol excretion failure. Secondly, infection-associated cellular injury may need extra cholesterol for new membrane synthesis and be beneficial to the host for protection from harmful effects of the stimuli [68]. The last assumption has good concordance with our 31P MRS data. The increased PME level has been hypothesized to be associated with intensified cell membrane synthesis [27,69] and regeneration [70,71]. The observed in our study correlations of PME and NTP-α peaks with the ALT, AST, cholesterol, HDL levels in serum and the cholesterol/phospholipid ratio in liver tissue of the infected hamsters supported the relation of these spectral parameters to liver injury and fibrosis.

However, there are some doubts concerning the power of the 31P MRS technique for assessing the degree of hepatic fibrosis [72]. Published literature has significant variations in the discovered correlation between 31P MRS peaks or its ratios and various liver abnormalities. Nonetheless a fibrosis marker, emphasized by the majority of authors, is the PME/PDE ratio; changes in the PME/PDE are thought to be associated with an increase in the regenerative efforts made by the damaged liver [73]. In our study we found neither changes in this ratio in *O*. *felineus* infection nor its correlation with fibrosis [72].

Thus, the correlation of NTP-α and PME peaks with fibrosis observed in our study can mainly reflect the metabolic changes in the liver, i.e. intensification of gluconeogenesis [74–76]. The assumption about intensification of gluconeogenesis in the liver in *O*. *felineus* infection is supported by the rise in PME as well as PME/NTP-α, PME/NTP-γ ratios in the infected group of hamsters. PME resonance, apart from PC and PE (that are believed to represent phospholipid cell membrane precursors), also includes gluconeogenesis intermediates, such as glucose-6-phosphate (G6P) and 3-phosphoglycerate (3PG) [27,69,74].

It is essential to describe the origin of the metabolic alteration. The critical role in local regulation of metabolism is played by the energetic status of the liver, especially the level and proportion of high-energy phosphates. In 31P MRS spectra, NTP-γ, -β and -α peaks are attributed to ATP, but also contain resonances from other triphosphates [77]. The NTP-β peak contains information almost exclusively from ATP [78]. In our experiment, the level of NTP-β as well as the Pi/NTP-β ratio in the liver of the infected hamsters did not change. Note that the ATP concentration in the liver of *O*. *felineus* infected animals, determined by the chemiluminescent assay, also maintained at a constant level. Thus, it may be concluded, that at this stage of infection the bioenergetics of the liver is not significantly disturbed.

Nonetheless, NTP-α and–γ peaks, apart from ATP, contain a contribution from ADP [77] in turn NAD+/NADP+ and NADH/NADPH resonances are the components of the NTP-α peak. 31P MRS data showed that Pi/NTP-α and Pi/NTP-γ as well as NTP-β/NTP-α ratios increased markedly in the infected group. Although 31P MRS did not allow us to make an accurate conclusion about the level of AMP and ADP, the observed spectral alteration indicated the changes in the high-energy phosphates ratio in the liver of the infected hamsters. In turn, this proportion plays a crucial role in cell metabolism regulation [79]. A drop in the AMP/ATP ratio results in dephosphorylation and inactivation of AMPK [49,80,81].

The decrease in the phospho-AMPK level in *O*. *felineus* infected liver and the correlation of the relative phospho-AMPK level with NTP-α peak intensity observed according to western blot analysis support our above-mentioned suggestions. So, when the AMP level is low and the ATP level is normal, glycolysis is nearly switched off and gluconeogenesis is promoted [82]. In turn, acetyl-CoA in the presence of adequate stores of ATP and low AMP levels is diverted to lipid synthesis [83]. Thus, lowering of the AMPK activity leads to a decrease in HMG-CoA reductase phosphorylation and promotes cholesterol synthesis [49]. Since acetyl-CoA carboxylase (ACC) is a substrate of AMPK, depletion of AMP increases the catalytic activity of ACC and the level of malonyl-CoA, which is a critical precursor for biosynthesis of fatty acids [49,80]. The invert correlations of the phospho-AMPK level with triglycerides and cholesterol serum concentration can be considered as an indirect evidence of lipid synthesis intensification in the infected animal liver. The mechanism underlying the decrease in the AMPK activity has to be further investigated, but it is important to note that, for example, TNF-α and IL-6 cytokines suppress AMPK [84,85].

At the system level, chronic liver injury and inflammation per se provoke chronic stress followed by a rise in the cortisol/corticosterone level. There are some publications about elevation of plasma cortisol in animals infected by parasitic worms [86,87]. This glucocorticoid(-s) has several effects on metabolism, in particular, it provokes mobilization of non-hexose substrates from extrahepatic tissues and stimulates synthesis of glucose in the liver. Simultaneously, chronic glucocorticoid excess is not associated with increased lipolysis in liver tissue; moreover, it may cause activation of hepatic lipogenesis [88,89]. What is more, determined in our study glycogen concentration in liver tissue of the experimental hamsters did not reduce during infection. Additionally, glucocorticoids demonstrate anti-inflammatory and immunosuppressive properties that can contribute to success of longstanding invasion of liver flukes. The contribution of cortisol/corticosterone to the observed metabolic disorder should be investigated further.

Apart from injury, inflammation and regeneration, the specific parasite-host interaction, i.e. molecules excreted by parasite adult worms during infection, may be the factor(s) leading to the observed metabolic changes [60,90]. In fact, the parasite’s goal is to take over the host, to provide comfortable conditions for its own habitat.

Arguably, *Opisthorchiidae* flukes are unable to synthesize cholesterol *de novo*. As an alternative, for example, *O*. *viverrini* has molecular pathways to acquire, transport and process cholesterol from external lipids [65]. Thus, biosynthesis of cholesterol by the host is very important for the parasite. Here the question arises, whether the parasite may evolve some opportunity (excreted molecular stimuli) to regulate the cholesterol metabolism of the host. Indeed, Adam et al. observed marked proliferation of smooth endoplasmic reticulum (ER) in hepatocytes of animals infected with *O*. *viverrini* [91]. In turn, enzymes in smooth ER are involved in lipid synthesis, including phospholipids and steroids.

For energy production the *Opisthorchiidae* fluke uses anaerobic and aerobic glycolysis [65]; during anaerobic glycolysis glucose is catabolized most likely to lactate. In spite of the fact that the parasite's genome encodes all of the tricarboxylic acid cycle enzymes living in microaerobic environment, it cannot effectively metabolize end-products of glycolysis along oxidative pathways and, therefore, they have to be excreted into the surrounding tissues of the host [92]. Further end-products of glycolysis may be utilized by the host liver in gluconeogenesis (preferable in the liver) or oxidative energy producing pathways. Recently in *in vitro* experiments it has been shown that excretory products of *O*. *viverrini* altered glycolysis/gluconeogenesis in normal human cholangiocytes (H69 cell line) [93]. The proposed changes of the high-energy phosphate ratios, as well as glucose metabolism, particularly, the gluconeogenesis rate, in *O*. *felineus* infected liver require additional investigation. However, the hypothesis about prevention of AMPK signaling activation by the parasite to retain the biosynthetic function of the liver, for the organ to produce cholesterol and glucose that are essential for liver fluke survival, seems not incurious.

Having analyzed the whole set of the obtained data, we may conclude that *O*. *felineus* infection disturbs hepatic metabolism of the host. The liver is considered to be a metabolic power station of mammalians, where cholesterol homeostasis relies on an intricate network of cellular processes, which deregulations can lead to severe pathologies [94]. In turn, the decreased AMPK activation, indicated in the infected hamster liver, is known to be implicated in metabolic disorders and associated with cancer risk [95]. Future investigations may shed light on the mechanism that leads to the observed metabolic changes and evaluate the contribution of *O*. *felineus* infection to known metabolic disorders. The knowledge about the capabilities of the parasite to regulate metabolism of the host may be applied for seeking a target for anti-helminth therapy and serve as the basis for developing new approaches to human metabolic disease regulation.

## Conclusion

The use of MR-techniques for investigating *O*. *felineus* infected liver has some advantages. MRI and MRS provide valuable information about the severity of liver impairments induced by opisthorchiasis. MR images of *O*. *felineus* infected liver have a characteristic pattern that differs from that of other liver fluke infections. The MRS findings point out the metabolic changes in the infected liver and identify the areas in which further scientific investigation is required. Further MR examination of infected humans allows clinicians to implement these methods in routine diagnosis of opisthorchiasis.

## Supporting information

S1 Text Blood and serum analysis. (DOCX)

S1 Table Laboratory parameters of the study cohort. (DOCX)

S2 Table Correlations between hepatic phosphorylated metabolites (^31^P MRS) and biochemical data of serum and liver tissue analysis. (DOCX)
