# Supplementary material for: Magnetic resonance imaging and spectroscopy for differential assessment of liver abnormalities induced by Opisthorchis felineus in an animal model
Source: PLoS Negl Trop Dis. 2017 Jul 14;11(7):e0005778. doi: 10.1371/journal.pntd.0005778 (PMC5529022; doi:10.1371/journal.pntd.0005778)
Supplement: S1 Text — (DOCX) [file pntd.0005778.s001.docx]

**S1 Text. Blood and serum analysis**

**Methods**

The blood samples were divided into two parts and placed in a red-top plastic tube containing a clot activator and a purple-top plastic tube containing [EDTA](https://en.wikipedia.org/wiki/EDTA). Serum analysis was performed on the biochemical analyzer Labio 200 (Mindray Medical International Ltd., China) using the following kits: Gamma GT (Vital Diagnostics, Italy), total bilirubin (Olvex, Russia), and glucose (DiaSys Diagnostic system GmbH, Germany). Aspartate aminotransferase (AST), alanine aminotransferase (ALT), alkaline phosphatase (ALP) and albumin were measured using kits purchased by Hospitex diagnostics (Sesto Fiorentino, Italy). Cholesterol, high-density lipoproteins (HDL), low-density lipoproteins (LDL), and triglycerides (TAG) were determined using Chronolab kits (Spain).

The hemoglobin concentration along with the white blood cell (WBC), the red blood cell (RBC) and the platelet count were determined using the Hematology Analyzer Hemolux-19 (Mindray Medical International Ltd., China). White blood cell types were counted manually by light microscopy.

**Results**

**S1 Table** provides the data on the biochemical parameters of blood and serum. The total blood cell count distinguished no significant differences in the amounts of WBC, RBC and platelets as well as the hemoglobin concentration in the control and infected groups. The blood differential test showed that the amount of eosinophils was elevated in the infected hamsters, whereas the monocyte count was lower than in the reference group. Eosinophilia is a common finding in parasitic infections. Eosinophilia in combination with monocytopenia may indicate an allergic reaction and depletion of body resources in response to chronic toxin released.

In the infected group of hamsters the level of ALT and gamma-glutamyl transferase (GGT) was markedly increased, whereas the ALP and AST levels did not show statistical differences among the groups. There was no difference in the serum bilirubin concentration in the infected and control animals.

The level of hepatic enzymes, except for ALP, correlated with the liver fibrosis stage determined according to the histological analysis. The calculated AST to ALT ratio (AAR) also showed strong correlation with the fibrosis stage. The 3-fold increase in the ALT level in serum and lowering of the AAR index <1 are highly associated with hepatocellular damage.

It is very interesting to note, that one hamster from the infected group had a very high level of ALP (1900 u L^-1^), but a relatively low (50 u L^-1^) ALT level. Markedly, this hamster had the most pronounced inflammation process (A2), according to the histological analysis performed in the infected group. Similar observation - very high ALP in combination with normal-to-low ALT – was made in our previous experiment in two hamsters from the experimental group infected for eight weeks (n=10, data not shown).

The concentration of albumin in serum of the infected hamsters was lower than in the reference group. The observed decrease in serum albumin was accompanied by a statistically significant increase in the urea concentration, however, there was no correlation between these parameters (r= -0.271, p=0.309).

The significant elevation of the cholesterol, triglyceride and the LDL concentrations in serum of the infected animals was detected.

There are contradictory data concerning the use of serum markers in opisthorchiasis diagnosis, so there are reports that either no difference or only slight elevation of the level of some liver enzymes occurs in the infection [[1-3](#_ENREF_1)]. Generally, a liver function test in human depends strongly on the stage of infection, invasion intensity as well as comorbidities.

The key blood marker of liver parenchymal cell injury is elevation of aminotransferases (ALT and AST). Both aminotransferases are highly concentrated in liver tissue. An increase in the serum ALT level is more specific for liver damage, and its release from liver tissue into the circulation is proportional to the degree of the hepatocellular damage [[4](#_ENREF_4)]. The elevated ALT level in serum of hamsters infected with *O. viverrini* and *O. felineus* was also observed in experiments by several authors [[5](#_ENREF_5),[6](#_ENREF_6)].

In the meantime, to diagnose biliary damage, ALP, GGT, and bilirubin are the most commonly used serum markers. However, Bulle et al. emphasized in their work, that the mechanism of increased activity of serum GGT differs from that of ALP activity elevation [[7](#_ENREF_7)]. The rise in the ALP and bilirubin levels results from acute biliary obstruction. ALP is associated with plasma membrane of hepatocytes adjacent to the biliary canaliculus and is absent in the cytoplasm of biliary cells. In case of cholestasis, not only ALP synthesis enhances, but also bile salt accumulates and mediates solubilization of membrane-bound enzymes. It results in release of hepatic ALP from the cell surface [[4](#_ENREF_4)]. Besides, excretion of bilirubin (product of hemoglobin degradation) with bile in the duodenum is impaired. On the contrary, an increase in the GGT level indicates bile duct damage as well as fibrosis, this enzyme being present both in biliary epithelial cells and in the cytoplasm of hepatocytes [[8](#_ENREF_8)]. GGT elevation in serum occurs earlier and persists longer than ALP increase in cholestatic disorders [[9](#_ENREF_9)], and this rise is associated with all forms of primary and secondary hepatobiliary pathologies. Elevations are moderate (2 to 5 times reference) with diffuse hepatic cell injury due to toxic or infectious hepatitis, whereas cholestasis due to intrahepatic or extrahepatic biliary obstruction causes 5 to 30 times increase in the GGT level in serum [[9](#_ENREF_9)]. In our study, GGT in serum of the infected hamsters rose only slightly (28 %). Thus, in case of opisthorchiasis at 8 weeks post-infection, the observed release of ALT and GGT from liver tissue into the circulation resulted from liver injury. The infection was accompanied by chronic cholangitis and fibrosis, but there was no evidence of bile duct occlusion.

The albumin concentration in serum of the infected hamsters markedly decreased. This can result from impaired protein secretion by damaged hepatocytes and/or intensification of protein catabolism. The assumption about intensification of protein catabolism stands in good agreement with the observed moderate azotemia. In fact, kidney injury and/or decreased blood flow through the kidneys cannot be excluded, however, moderate elevation of the urea level in serum can be caused by raised production of urea in the liver via increased protein catabolism [[10](#_ENREF_10)]. Such processes as injury and inflammation provoked by infection are often accompanied by protein catabolism intensification and increased urinary nitrogen excretion [[11](#_ENREF_11)].

**References**

1. Sripa B, Mairiang E, Thinkhamrop B, Laha T, Kaewkes S, et al. (2009) Advanced periductal fibrosis from infection with the carcinogenic human liver fluke Opisthorchis viverrini correlates with elevated levels of IL-6. Hepatology 50: 1273-1281.

2. Armignacco O, Caterini L, Marucci G, Ferri F, Bernardini G, et al. (2008 ) Human Illnesses Caused by Opisthorchis felineus Flukes, Italy. Emerg Infect Dis 14: 1902-1905.

3. Feldmeier H, Hazay M, Sato M, Tiengkham P, Nishimoto F, et al. (2016) Morbidity assessment of Opisthorchis viverrini infection in rural Laos: I. Parasitological, clinical, ultrasonographical and biochemical findings. Trop Med Health 44: 12.

4. Giannini EG, Testa R, Savarino V (2005) Liver enzyme alteration: a guide for clinicians. CMAJ 172: 367-379.

5. Boonjaraspinyo S, Boonmars T, Aromdee C, Srisawangwong T, Kaewsamut B, et al. (2009) Turmeric reduces inflammatory cells in hamster opisthorchiasis. Parasitol Res 105: 1459-1463.

6. Maksimova GA, Pakharukova MY, Kashina EV, Zhukova NA, Lvova MN, et al. (2016) The morphofunctional and biochemical characteristics of opisthorchiasis-associated cholangiocarcinoma in a Syrian hamster model. Russ J Genet Appl Res 6: 454.

7. Bulle F, Mavier P, Zafrani ES, Preaux AM, Lescs MC, et al. (1990) Mechanism of gamma-glutamyl transpeptidase release in serum during intrahepatic and extrahepatic cholestasis in the rat: a histochemical, biochemical and molecular approach. Hepatology 11: 545-550.

8. Irie M, Suzuki N, Sohda T, Anan A, Iwata K, et al. (2007) Hepatic expression of gamma-glutamyltranspeptidase in the human liver of patients with alcoholic liver disease. Hepatol Res 37: 966-973.

9. Vroon DH, Israili Z (1990) Alkaline Phosphatase and Gamma Glutamyltransferase. In: Walker HK, Hall WD, Hurst JW, editors., editors. Clinical Methods: The History, Physical, and Laboratory Examinations 3rd edition. Boston: Butterworths.

10. Bishop ML, Fody EP, Schoeff LE (2010) Clinical Chemistry: Techniques, Principles, Correlations. 6th Edition. Baltimore, PA: Lippincott Williams and Wilkins.

11. Wilmore DW (1999) Infection and Injury: Effects on Whole Body Protein Metabolism. Protein and Amino Acids. Washington, D.C.: National Academy Press. pp. 155-167.
