# Supplementary material for: Magnetic resonance imaging and spectroscopy for differential assessment of liver abnormalities induced by Opisthorchis felineus in an animal model
Source: PLoS Negl Trop Dis. 2017 Jul 14;11(7):e0005778. doi: 10.1371/journal.pntd.0005778 (PMC5529022; doi:10.1371/journal.pntd.0005778)
Supplement: S1 Table — (DOCX) [file pntd.0005778.s002.docx]

**S1 Table. Laboratory parameters of the study cohort**

| Parameter | Reference^#^ | | Control | | Infected | | Correlation to fibrosis stage | |
| --- | --- | --- | --- | --- | --- | --- | --- | --- |
|  | mean | SD | mean | SD | mean | SD | r | p-level |
| Liver to body weight ratio, % | ND | | 2.72 | 0.28 | 3.80** | 0.74 | - | - |
| Spleen to body weight ratio, % | ND | | 0.17 | 0.26 | 0.14 | 0.09 | - | - |
| **Blood** | | | | | | | | |
| WBC, 10^9/L | 7.60 | 1.30 | 3.98 | 1.92 | 8.24 | 7.09 | 0.495 |  |
| Banded neutrophils, % | 8.00 | 2.50 | 1.25 | 0.50 | 1.00 | 0.00 | -0.330 | 0.211 |
| Segmented neutrophils, % | 22.10 | 2.50 | 31.13 | 9.33 | 34.88 | 17.34 | -0.006 | 0.981 |
| Eosinophils, % | 0.90 | 0.30 | 1.67 | 1.03 | 7.88** | 3.64 | 0.871 | 0.000 |
| Basophils, % | 1.00 | 0.20 | 0 | 0 | 0 | 0 | - | - |
| Monocytes, % | 2.50 | 0.80 | 64.00 | 3.70 | 52.75 | 5.50 | -0.730 | 0.001 |
| Lymphocytes, % | 73.50 | 9.40 | 4.25 | 0.59 | 6.25** | 0.67 | 0.333 | 0.208 |
| RBC, 10^12/L | 7.50 | 1.40 | 8.30 | 0.42 | 7.83 | 1.52 | -0.104 |  |
| Hemoglobin, g L^-1^ | 168.00 | 12.00 | 162.60 | 9.94 | 150.00 | 26.02 | -0.091 |  |
| Platelets, 10^9/L | ND | | 385.40 | 183.30 | 446.80 | 167.50 | 0.202 |  |
| **Serum** | | | | | | | | |
| Albumin, g L^-1^ | 43.00 | 2.20 | 39.85 | 1.29 | 37.74* | 2.20 | -0.396 | 0.129 |
| ALT, u L^-1^ | 38.00 | 26.10 | 85.00 | 25.31 | 363.80** | 153.40 | 0.661 | 0.005 |
| AST, u L^-1^ | 47.00 | 38.30 | 95.75 | 13.67 | 138.90 | 59.39 | 0.499 | 0.049 |
| GGT, u L^-1^ | ND | | 5.88 | 0.35 | 7.50* | 1.77 | 0.703 | 0.002 |
| ALP, u L^-1^ | 218.00 | 42.00 | 319.00 | 18.91 | 509.60 | 562.50 | -0.050 | 0.853 |
| Total bilirubin, µmol L^-1^ | 3.42-12.66^@^ | | 3.95 | 3.03 | 3.09 | 2.22 | -0.280 | 0.294 |
| Total cholesterol, mmol L^-1^ | 2.90-5.43^@^ | | 2.96 | 0.34 | 4.30** | 0.84 | 0.697 | 0.003 |
| HDL, mmol L^-1^ | ND | | 0.75 | 0.39 | 1.19 | 0.64 | 0.295 | 0.266 |
| LDL, mmol L^-1^ | ND | | 0.49 | 0.11 | 1.32** | 0.61 | 0.713 | 0.002 |
| Triglycerides, mmol L^-1^ | 0.82-2.60^@^ | | 1.43 | 0.33 | 1.98** | 0.26 | 0.652 | 0.006 |
| Glucose, mmol L^-1^ | 4.05 | 0.72 | 4.48 | 1.19 | 4.56 | 1.04 | -0.080 | 0.769 |
| Urea, mmol L^-1^ | 3.16 | 0.67 | 5.71 | 0.31 | 6.68* | 1.09 | 0.538 | 0.031 |
| AAR | ND | | 1.19 | 0.26 | 0.48** | 0.32 | -0.778 | 0.000 |

*difference between the infected and the control group is significant at p˂0.05.

**difference between the infected and the control group is significant at p˂0.01

^#^ parameter values taken from (Van Hoosier, G.L., McPherson, C.W., 1987. Laboratory hamster. Academic Press Inc., New York)

^@^ data are given as range, parameter values taken from (Field, K.J., Sibold, A.L., 1998. The Laboratory Hamster and Gerbil. CRC Press, Boca Raton)

Abbreviation: ND – no data; r, Correlation coefficient; ALT, Alanine transaminase; AST, Aspartate aminotransferase; GGT, Gamma-glutamyltransferase; ALP, Alkaline phosphatase; HDL, High-density lipoproteins; LDL, Low-density lipoproteins; AAR, Aspartate aminotransferase /alanine aminotransferase ratio.
