# Supplementary material for: Magnetic resonance imaging and spectroscopy for differential assessment of liver abnormalities induced by Opisthorchis felineus in an animal model
Source: PLoS Negl Trop Dis. 2017 Jul 14;11(7):e0005778. doi: 10.1371/journal.pntd.0005778 (PMC5529022; doi:10.1371/journal.pntd.0005778)
Supplement: S2 Table — (DOCX) [file pntd.0005778.s003.docx]

**S2 Table. Correlations between hepatic phosphorylated metabolites (^31^P MRS) and biochemical data of serum and liver tissue analysis**

|  | Parameter | PME | | Pi | | PDE | | NTP-γ | | NTP-α | | NTP-β | |
| --- | --- | --- | --- | --- | --- | --- | --- | --- | --- | --- | --- | --- | --- |
|  |  | r | p-level | r | p-level | r | p-level | r | p-level | r | p-level | r | p-level |
| **Serum** | Albumin | -0.258 | 0.335 | -0.240 | 0.370 | 0.113 | 0.678 | 0.334 | 0.207 | 0.273 | 0.307 | -0.279 | 0.296 |
|  | ALT | 0.830 | 0.000 | 0.615 | 0.011 | 0.435 | 0.092 | -0.215 | 0.424 | -0.813 | 0.000 | 0.055 | 0.840 |
|  | AST | 0.580 | 0.019 | 0.560 | 0.024 | 0.375 | 0.153 | -0.047 | 0.861 | -0.543 | 0.030 | -0.284 | 0.286 |
|  | GGT | 0.057 | 0.835 | 0.065 | 0.810 | 0.134 | 0.621 | -0.183 | 0.498 | -0.307 | 0.247 | 0.404 | 0.121 |
|  | ALP | -0.197 | 0.465 | -0.282 | 0.289 | -0.407 | 0.118 | 0.088 | 0.745 | 0.119 | 0.659 | 0.276 | 0.301 |
|  | Total bilirubin | -0.122 | 0.653 | -0.292 | 0.273 | 0.095 | 0.725 | -0.057 | 0.834 | 0.281 | 0.291 | -0.192 | 0.477 |
|  | Total cholesterol | 0.629 | 0.009 | 0.226 | 0.400 | 0.173 | 0.523 | -0.283 | 0.288 | -0.644 | 0.007 | 0.080 | 0.769 |
|  | HDL | 0.775 | 0.000 | 0.342 | 0.195 | 0.299 | 0.260 | 0.083 | 0.759 | -0.591 | 0.016 | -0.423 | 0.102 |
|  | LDL | 0.493 | 0.052 | 0.191 | 0.480 | -0.032 | 0.906 | -0.090 | 0.714 | -0.492 | 0.053 | 0.101 | 0.709 |
|  | Triglycerides | 0.366 | 0.163 | 0.047 | 0.862 | -0.030 | 0.913 | -0.244 | 0.363 | -0.492 | 0.053 | 0.589 | 0.016 |
|  | Glucose | -0.121 | 0.655 | -0.153 | 0.571 | 0.349 | 0.185 | -0.075 | 0.782 | 0.083 | 0.759 | -0.216 | 0.421 |
|  | Urea | 0.325 | 0.219 | 0.208 | 0.441 | 0.246 | 0.359 | 0.078 | 0.774 | -0.421 | 0.105 | 0.003 | 0.991 |
|  | AAR | -0.736 | 0.001 | -0.524 | 0.037 | -0.478 | 0.061 | 0.230 | 0.391 | 0.692 | 0.003 | -0.151 | 0.576 |
| **Liver**  **tissue** | Protein, mg g^-1^ | -0.254 | 0.343 | -0.174 | 0.518 | -0.217 | 0.419 | 0.179 | 0.506 | 0.364 | 0.165 | -0.172 | 0.524 |
|  | Triglycerides | -0.054 | 0.842 | 0.102 | 0.706 | -0.620 | 0.010 | 0.022 | 0.936 | 0.101 | 0.708 | 0.614 | 0.011 |
|  | Total cholesterol | 0.344 | 0.192 | 0.400 | 0.125 | -0.193 | 0.473 | -0.240 | 0.370 | -0.375 | 0.152 | 0.606 | 0.013 |
|  | Phospholipid | -0.510 | 0.043 | -0.376 | 0.151 | -0.364 | 0.165 | -0.099 | 0.715 | 0.448 | 0.082 | 0.183 | 0.496 |
|  | Total lipid | -0.116 | 0.670 | -0.038 | 0.890 | -0.567 | 0.022 | 0.208 | 0.439 | 0.498 | 0.050 | 0.048 | 0.860 |
|  | Triglycerides to phospholipid ratio | 0.134 | 0.622 | 0.266 | 0.319 | -0.547 | 0.028 | 0.070 | 0.797 | -0.049 | 0.856 | 0.612 | 0.012 |
|  | Cholesterol to phospholipid ratio | 0.548 | 0.028 | 0.489 | 0.055 | -0.034 | 0.901 | -0.229 | 0.394 | -0.554 | 0.026 | 0.508 | 0.045 |
|  | ATP | -0.131 | 0.627 | -0.246 | 0.359 | -0.129 | 0.635 | -0.201 | 0.455 | 0.128 | 0.638 | -0.055 | 0.840 |
|  | Phospho-AMPK, % of beta-actin | -0.471 | 0.138 | -0.049 | 0.867 | -0.233 | 0.423 | 0.272 | 0.348 | 0.586 | 0.028 | -0.057 | 0.847 |

Abbreviations: r, Correlation coefficient; ALT, Alanine transaminase; AST, Aspartate aminotransferase; GGT, Gamma-glutamyltransferase; ALP, Alkaline phosphatase; HDL, High-density lipoproteins; LDL, Low-density lipoproteins; ATP, adenosine triphosphate; Phospho-AMPK, 5’-AMP-activated protein kinase phosphorylated by Thr172 residue of α-subunit; PME, Phosphomonoesters; Pi, Inorganic phosphate; PDE,
